# Adolescent Preferences and Design Recommendations for an Asthma Self-Management App: Mixed-Methods Study

**DOI:** 10.2196/10055

**Published:** 2018-09-13

**Authors:** Courtney Roberts, Adam Sage, Lorie Geryk, Betsy Sleath, Delesha Carpenter

**Affiliations:** 1 Division of Pharmaceutical Outcomes and Policy Eshelman School of Pharmacy University of North Carolina at Chapel Hill Chapel Hill, NC United States; 2 Center for Health Systems Effectiveness Oregon Health and Sciences University Portland, OR United States; 3 The Cecil G Sheps Center for Health Services Research University of North Carolina at Chapel Hill Chapel Hill, NC United States; 4 Division of Pharmaceutical Outcomes and Policy Eshelman School of Pharmacy University of North Carolina at Asheville Asheville, NC United States

**Keywords:** asthma, mHealth, mobile app, patient engagement, self-management, usability

## Abstract

**Background:**

Approximately 10% of adolescents in the United States have asthma. Adolescents widely use apps on mobile phones and tablet technology for social networking and gaming purposes. Given the increase in recreational app use among adolescents, leveraging apps to support adolescent asthma disease management seems warranted. However, little empirical research has influenced asthma app development; adolescent users are seldom involved in the app design process.

**Objective:**

The aim of this mixed-methods study was to assess adolescent preferences and design recommendations for an asthma self-management app.

**Methods:**

A total of 20 adolescents with persistent asthma (aged 12-16 years) provided feedback on two asthma self-management apps during in-person semistructured interviews following their regularly scheduled asthma clinic visit and via telephone 1 week later. Interviews were audiorecorded, transcribed verbatim, analyzed using SPSS v24, and coded thematically using MAXQDA 11.

**Results:**

Regarding esthetics, app layout and perceived visual simplicity were important to facilitate initial app use. Adolescents were more likely to continually engage with apps that were deemed useful and met their informational needs. Adolescents also desired app features that fit within their existing paradigm or schema and included familiar components (eg, medication alerts that appear and sound like FaceTime notifications and games modeled after Quiz Up and Minecraft), as well as the ability to customize app components. They also suggested that apps include other features, such as an air quality tracker and voice command.

**Conclusions:**

Adolescents desire specific app characteristics including customization and tailoring to meet their asthma informational needs. Involving adolescents in early stages of app development is likely to result in an asthma app that meets their self-management needs and design preferences and ultimately the adoption and maintenance of positive asthma self-management behaviors.

## Introduction

In the United States, 1 in 10 adolescents have asthma [1], and poorly controlled asthma among children and adolescents (aged 6-17 years) can lead to greater health resource utilization and health care expenditures estimated at US $5.35 billion per year [2]. However, negative health outcomes can be drastically reduced and prevented through proper disease self-management [3], and leveraging technology to support adolescent asthma management seems warranted. A little over half (58%) of adolescents aged 13-17 years have access to a tablet, approximately 75% have access to a smartphone, and 92% of teens report going online daily [4].

Although adolescents are early adopters of technology and mobile app use is increasing among the adolescent population, only 8 out of 147 asthma apps are targeted toward children or young adults [5]. Given that declines in treatment adherence have been observed during adolescence and adolescents with asthma are at higher risk for poor health outcomes than other age groups with asthma [6-8], apps targeting this age group are crucial. However, a challenge for health apps compared with popular mainstream apps is their lack of use over time considering that many health-related apps are cumbersome and uninteresting to the app user [9]. Research has found that although 21% of teens have downloaded a health-related app, only 8% of these teens often used the app and 47% hardly ever or never used them [10]. Qualitative research on adolescent preferences for health apps, including how they perceive and would use apps to support their asthma self-management, has been lacking [11,12].

The objective of our study was to assess adolescent preferences and design recommendations for an asthma self-management app. We obtained feedback from adolescents with asthma on 2 existing asthma self-management apps and examined preferences for esthetics, features, and app content that would encourage continued engagement with the app. In addition, we assessed adolescents’ overall opinions of the apps, focusing on aspects of app usability. Understanding adolescent preferences and reasons for suggested changes and design recommendations could lead to the development of an app with better usability, user engagement, and improved clinical outcomes.

## Methods

### Study Population and Recruitment

A total of 20 adolescents were recruited from 2 pediatric medical practices in urban North Carolina. Eligible adolescents were (1) 12-16 years of age; (2) able to read and understand English; (3) owned a cell phone, smartphone, or tablet; (4) present at the medical visit with an adult caregiver; and (5) had persistent asthma (defined as asthma-related daytime symptoms >2 times a week and nighttime symptoms >2 times a month or receiving ≥1 long-term asthma control therapies) [13,14]. A clinic liaison used the electronic health record to identify potentially eligible adolescents with asthma at both pediatric practices and informed their caregivers about the study over the phone prior to the adolescent’s regularly scheduled clinic appointment. When they arrived for their appointment, interested adolescents and their caregivers were introduced to the study research assistant and were screened for eligibility. Participating adolescents provided written informed assent, and caregivers provided parental permission for their child to participate.

### Study Procedures

Adolescents completed a brief demographic questionnaire prior to their clinic visit. After their visit, the research assistant gave adolescents an iPod with 2 preloaded asthma self-management apps. The two apps were purposively selected by a behavioral researcher, an mHealth expert, and a respiratory therapist from those available on the iOS platform due to their user-friendly interface and inclusion of features for asthma self-management. Since no apps at the time of the study (pre-2017) were targeted specifically for adolescents, we selected one app geared toward children and one for adults. The research assistant reviewed the features of the apps with the adolescents and their caregiver, and then let them explore the apps on their own for approximately 10 minutes. After adolescents were familiar with the apps, adolescents and caregivers provided their feedback during a semistructured 30-minute interview. Adolescents were asked to use the apps over the next 7 days, after which they completed a 30-minute telephone interview that further assessed their app preferences and design recommendations. In-person and telephone interviews were both audiorecorded. iPod touches were given to the adolescents as study incentives. The study was reviewed and approved by the Institutional Review Board at the University of North Carolina and was conducted in accordance with the tenets of the Declaration of Helsinki.

### Measures

#### Sample Demographic and Clinical Characteristics

Adolescents reported the following demographic characteristics: (1) gender (male or female); (2) age (in years); (3) ethnicity (Hispanic, Latino or Spanish origin); and (4) race (white, black or African American, American Indian or Alaskan Native, Asian, Native Hawaiian or Other Pacific Islander, or other), which was recoded into 3 variables (white, black or African American, or other) for descriptive purposes. Adolescents also reported their grade in school, how long they have had asthma (in years), and how serious they think their asthma is (1=very serious, 2=fairly serious, 3=somewhat serious, and 4=not at all serious).

#### Technology Use

Adolescents were asked the following yes or no questions:

1. Have you downloaded an app to a cell phone, tablet, or other handheld device?
2. Have you ever downloaded a health-related app to a cellphone, tablet, or other handheld device?
3. Have you ever used an asthma app before today?
4. Do you enjoy spending time on social internet sites such as Twitter, Instagram, Facebook, or Pinterest?

Participants were also asked, “Which site(s) do you use most of the time?”, and were able to select ≥1 of the sites above or select “other” and list the other site used most of the time.

### Interview Content

Adolescent app preferences and design recommendations were assessed using a process of qualitative inquiry [15]. Researchers employed an evaluation methodology to ascertain adolescents’ feedback on the apps during both in-person visit and 1-week follow-up telephone interview [16]. During the initial in-person interview, adolescents were asked questions focusing on the visual appeal of the apps, suggestions for improvement (eg, additions and changes), and overall feedback on the apps. They were also asked about their opinions on the apps after exploring them for a week, including ease of use, barriers and facilitators to use, and additional suggestions for improvement.

Interview questions that assessed adolescent interest in additional components like asthma quizzes, avatars, and videos for inhaler technique, as well as questions pertaining to preferred app and app self-management potential, were quantified (yes or no). Table 1 lists the relevant interview questions that were used to assess adolescent preferences and design recommendations.

### Data Analysis

IBM SPSS version 24 (Armonk, New York) was used to calculate descriptive statistics and analyze quantitative responses, and MAXQDA version 11 (Berlin, Germany) was used to analyze qualitative interview data. Both in-person and telephone interviews were transcribed verbatim, deidentified, and analyzed thematically by 3 research team members. These team members engaged in an iterative process of reading and rereading the transcripts to identify relevant themes and create a detailed coding tool [17,18]. This coding tool included code definitions and example quotations to help improve interrater reliability. A process of open coding and axial coding was utilized to sort data into topical categories, identify emergent themes, and examine relationships among themes [18]. Table 1 lists the relevant interview questions, as well as whether these questions were asked during the in-person or follow-up interviews.

**Table 1:** Adolescent preferences and design recommendations guide.

Coding category		Interview questions	
**Qualitatively analyzed questions**			
	Ease of use		Can you please describe what it was like learning how to use these apps?^a^ How much time would you say that it took you to learn how to use (preferred app)?^a,b^
	App engagement/barriers and facilitators to use		Do you think you will continue to use (preferred app)? Why or why not?^a^ Can you tell us the reason or reasons that you did not use the applications?^a^ Can you think of anything that would have helped you use the applications?^a^
	Overall feedback		Is there anything else that we haven’t covered about the apps that you would like to share?^c^
	Suggestions for improvement		How could (child app) be changed to make it better?^c^ How could (adult app) be changed to make it better?^c^ What would you add that you didn’t see as a feature—in either app—and why?^c^ How could any of these features be improved?^a^ Is there a feature that you think was missing in this app that you would like to use?^a^ What would you change about (preferred app)?^a^
	Visual appeal		Which app do you think looks better and why?^b,c^
**Quantitatively analyzed questions**			
	Asthma quizzes		Would you take asthma quizzes if they were part of the app?^a^
	Avatar		Would you be more likely to use an asthma app if you could create an avatar?^a^
	Inhaler technique		Would you use the app to watch videos of correct inhaler technique?^a^
	Preferred app		Using a scale of 1-5, 1 being not at all satisfied and 5 being extremely satisfied, please rate your overall satisfaction with (app name). (App with higher score=preferred app)
	Self-management potential		Which app helped you most with managing your asthma?^a^

^a^Telephone interview.

^b^Quantified for analysis.

^c^In-person interview.

All audiorecorded transcripts were coded independently by one research team member, and a secondary coder coded 10% of all transcripts. Interrater reliability was kappa=.85.

## Results

### Adolescent Demographic Characteristics

The demographic characteristics of adolescents who participated in our study are shown in Table 2. Among them, 20 completed the in-person interview and 16 completed the 1-week follow-up phone interviews. The demographic characteristics of the 4 adolescents who did not complete the phone interviews did not differ from those who completed the study (2 white, 2 black, and 3 females with a mean age of 14.5 years).

### Adolescent Technology Usage

Although all 20 adolescents had previously downloaded an app, only 15% (3/20) had downloaded a health-related app and none had used an asthma app before the study. Moreover, 75% (15/20) adolescents said that they enjoyed spending time on social media, and the site(s) that adolescents reported using most of the time were Facebook (11/20, 55%), Instagram (8/20, 40%), Twitter (5/20, 25%), and Pinterest (3/20, 15%).

### App Ease of Use

The minimum time an adolescent spent learning how to use the apps was 30 seconds, with one adolescent stating it took them “a couple of minutes” and another one describing the time as “not long.” About a third of the adolescents from the follow-up interviews (5/16, 31%) spent approximately 5-10 minutes learning how to use the apps, and others reported it took them longer—1-3 days (the maximum time reported) (6/16, 38%).

App ease of use was an important facilitator of adolescent engagement with the app and continual use over the 7-day period. When asked, “Do you think you will continue to use [preferred app], and why?”, one adolescent responded that she would continue to use the app.

It’s very simple and easy to use…you just go in and put in a couple of things [in the app] instead of a really long process because I think once you have too many features on it, you know, you don’t want to use it every single day.

Female, 14

While some adolescents stated that learning how to use the apps was “pretty easy for the most part” because “there wasn’t much to them, they’re kind of self-explanatory,” others expressed some difficulty.

It was easy and it was a little confus-, well, it wasn’t confusing, it was just—I had to learn how to use it, because I’m not—you know, I’m not used to using the app—well, I am now, but I wasn’t at the time, so I had to learn how to use it and I had put information in it.

Female, 15

Like I mean like to understand it real well and to know how to use it, like I had to get used to it cause I never seen another application like this one.

Male, 14

One adolescent stated that, “it doesn’t explain how to use it well.” Adolescents reported unfamiliarity with the apps and a lack of understanding on how to use the apps as barriers to initial app use. However, adolescents suggested improvements to the apps that would aid in their app understanding, including video tutorials and picture explanations, and increase their app ease of use.

Maybe it could give you like a list of it, like a tutorial before you like actually started using it…to like show you through the app and what the stuff is.

Male, 12

[Add] Videos of people actually like using it, you know, like a tutorial walk through. And also, like the things like also showing like how some people are using it, like some videos of people using it and getting used to it.

Male, 12

Add pictures to [app name]. Because I think if you were to add that, I think more teenagers my age could understand the app better because they don’t understand what it is.

Male, 14

**Table 2:** Adolescent sample characteristics (N=20).

Adolescents		Value
Age in years (range: 12-17), mean (SD)		14.7 (1.6)
Female, n (%)		9 (45)
**Race, n (%)**		
	White	9 (45)
	Black	8 (40)
	Other	3 (15)
**Ethnicity, n (%)**		
	Hispanic, Latino, or Spanish origin	4 (20)
	Non-Hispanic	16 (80)
Grade in school (range: 6-11), mean (SD)		8.3 (1.6)
Years with asthma (range: 1-16), mean (SD)		9.9 (4.8)
Perceived asthma severity (range: 1-4), mean (SD)		2.4 (0.9)

### App Visual Appeal

**Figure 1:**
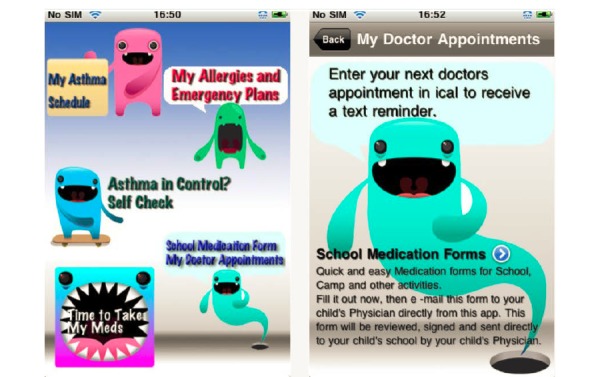
Screenshots from the child app. Source: iAsthma.

**Figure 2:**
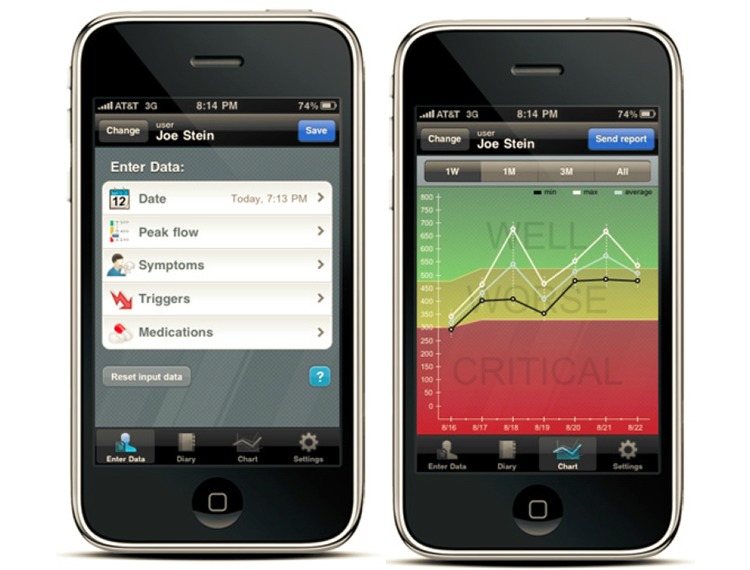
Screenshots from the adult app. Source: AsthmaMD.

Figures 1 and 2 depict screen shots taken from the apps. Adolescents expressed mixed opinions regarding the visual appeal of the two apps. Referencing the child app, adolescents commented the following:

I like how like it’s all like colorful and like playful and like looks like it’s real—like it looks interesting, you know.

Male, 12

[Child app] made me smile when I used it, I don’t know. It’s kind of funny, like every time you click, and you know how it made that noise? Yeah it was pretty cool…that makes me want to interact with it more.

Male, 16

They stated that the app was “interactive for kids,” and they liked that it was simplistic—citing the fact that it does not have too many features as a plus. They also liked that all the main features were visible on one main page, so it “has everything like right there so like you can just click on it” [Male, 12]. However, others stated:

It’s kind of kid-is to me, it’s more for younger kids, not really for teens…it seemed like it was more for kids that were like 12 and under.

Male, 15

The monsters and stuff that it has, I personally don’t like it.

Female, 16

Some also negatively commented on the design layout, mentioning that things were “all clumped on one page,” and “cluttered…and need to be split up.”

Regarding the adult app, some participants stated that “It’s more organized [than the child app]” [Female, 16], and they liked that the app labels everything. However, others stated:

[The adult app] has so much stuff in it, and I don’t really know how to use all that stuff just quite yet

Male, 13

[It looks] really complex…[developers need to] make it a little more appealing to kids

Male, 13

Despite mixed reviews regarding which app was more visually appealing and organized, app layout and perceived visual simplicity were important to adolescents. When asked, “How could [preferred app] be changed to make it better?”, one adolescent stated that they would like to see the app organized differently, with the main features in an alphabetical order. Adolescents also enjoyed visual aids, such as pictures, graphs, and charts, stating:

They just work that you might see or want to learn with maybe, like the charts, stuff like that.

Male, 14

That [chart] was what really…helped me because you could really visualize how your asthma is doing.

Female, 14

### Qualitative Themes

Three qualitative themes emerged from the interviews.

#### Customization to Fit Within the Schema

Adolescents suggested feature-specific customizations, such as adding more preselected symptoms, triggers, and medications to the medication list, as well as the option to input your own.

They also proposed improvements to the interface, including the option to have bigger icons, as well as changes to text, background, graphics, and pictures. One adolescent stated that their preferred app “would keep someone’s interest, because with all the colors and the characters and stuff” [Female, 16]. However, many agreed that:

It might be cool if you could choose like your colors and stuff on it…if you wanted to choose like a theme color.

Female, 14

Several adolescents criticized the apps stating the following:

The graphics weren’t like good enough, like for a teen to like want to use it…it would just like make them feel like they’ve downloaded it so like they…have to use it because they have like a, um, certain situation, it won’t make them like want to use it, it’ll just like, you know, make them feel like they have to use it.

Male, 12

The main menu looks…kind of hard to read because of the shadow, um it looks kind of like PowerPoint, a strange PowerPoint slide. I feel like the shadow in the background does not need to be there…it looks like someone took a lot of the word art things that you can do and then put them all there. It looks like I could probably go and make something very similar.

Female, 14

The words are kind of a little blurry, but like I know they try their best, I give them props and stuff for that cause I can’t do that, and they did good, but the graphics [are] just like a little off and like it’s kind of blurry.

Male, 13

One adolescent pointed out that the app might not look the same on the new version of the iPhone, stating the new iPhone “has a different kind of interface, so it might look a little outdated on those.”

It seems to look like the iOS, it doesn’t look like the new operating system. So, it might be nice if it could look different for the different type of like devices because I know the, the iPhones, my friend has one, it’s got a whole new like look to it. Catch up with that one.

Female, 14

Adolescents specifically stated app customizations that seemed to fit within their current schema or apps and technology with which they were already familiar. For example, one adolescent suggested the addition of a medication reminder that would…

…pop up on your screen with an—with, uh, your like ringtone for maybe FaceTime or something…You would have to set a ringtone through the app, though, but it would like—your phone would either buzz—your iPod or phone or whatever you have would buzz and it would just like remind you to take the medicine.

Male, 12

One adolescent stated they would be attracted to an app if it could incorporate “something that teens are interested in…like there’s a game that like most of us play, called Minecraft” [Male, 12]. He suggested modeling an asthma game after Minecraft, and another one similarly suggested modeling a game after QuizUp to facilitate app engagement.

I know like that app, the QuizUp is like really fun, even though it’s like quiz stuff, it’s fun to do because you’re like competing against other people. And yeah, I know a lot of people who like that game, so, if you could like do basically something like that, except with asthma stuff, I think it’d be fun.

Female, 16

When asked, “How could any of these features be improved?”, one adolescent responded by mentioning adding puzzle games because she plays those on other apps, and many adolescents suggested the addition of asthma games or turning existing features into gaming components since “kids love games” [Female, 13]. For example, one adolescent stated, “if there was a cool game on it that interpreted what the asthma stuff [means], then I guess I’d use it,” and another one stated “Like if there was like a game on the app, I think kids would enjoy it better” [Female, 13]. An adolescent suggested a game idea below:

Let’s say like a normal patient has asthma and they had to take their inhaler, sort of a few puffs day and night, so like you could set a notification for like 12 hours for them to take their medication. And like if, like if you want to make it like a little more good to the kid population, like you could say, you know, you took your medication, 50 points or something.

Male, 13

One adolescent stated that he would like to see more…

…pictures of stuff that like inspire you. Like for me, I like sports, so I would like to see like sports people or, uh, game characters and stuff like that.

Male, 14

Similarly, one adolescent stated that he would add Cam Newton or his favorite sports character and incorporate him into the app, possibly as an avatar, or include an image of him on a page of the app. When asked, would you be more likely to use an asthma app if you could create an avatar?”, 63% (10/16) said yes, 6% (1/16) said most likely, 12% (2/16) said probably, and 19% (3/16) said no.

#### Utility or Functionality of Apps Over Visual Appeal for Continual Engagement

Although adolescents stressed that an app should be visually appealing in order to facilitate app use, many adolescents appeared to place equal to higher importance on app functionality and self-management potential over app appearance for continual engagement. Of the 16 adolescents who completed the follow-up interview, 63% (10/16) said the child app looked better, while 31% (5/16) said the adult app looked better (1 adolescent did not respond). However, despite majority preference for the visual appeal of the child app, only 31% (5/16) of these adolescents said the child app was their preferred app compared with 69% (11/16) adolescents citing the adult app as their preferred app. Furthermore, 63% (10/16) adolescents said the adult app was best for managing their asthma compared with 25% (4/16) adolescents for the child app (2 did not respond).

One adolescent stated the child app was “too basic” with its one primary page layout. She stated that “the [adult app] has more pages to choose from and you can find out more things, like going on the website and asking questions” [Female, 16]. When it came to meeting asthma self-management needs, the child app was deemed “less comprehensive” with adolescents stating:

I like the graphics, they’re cute, but, um, it doesn’t—it doesn’t seem like it would be as useful.

Female, 14

I don’t understand the point of it, personally. Um, so do you—when you take your meds do you just drag it into the mouth?…I didn’t under—like I didn’t get the point of it, but, I mean, it’s cute, but I didn’t get the point.

Female, 16

Similarly, some adolescents stated that they liked the adult app because “it covers more stuff” [Female, 14] and “it was very useful… It looks like it helps more, and it takes it [asthma] more serious and stuff” [Male, 14]. They noted the wide range of self-management features available in the adult app, citing:

It [adult app] has more things that are like useful to like track the stuff that you are entering than the [child app].

Male, 12

One adolescent stated she would…

…just use the monster one [child app] to look at the monsters, but… I would use the other one to like keep track of like how my asthma was that—like that day, like if it got real bad.

Female, 13

Similarly, another adolescent stated:

The [child app] is more attractive and the [adult app] is more like functional.

Female, 14

#### Tailoring the App to Meet Asthma Informational Needs

Adolescents really wanted app features that met their asthma self-management and informational needs. For example, one adolescent suggested that if the app had more information where…

…if something bad happened, like if something triggered my asthma, and then I wanted to like find out more about it and how I could get better and like why my asthma flared up or whatever, so if it had that, then I’d use it [the app].

Female, 16

Adolescents suggested improvements to the apps that would aid in their self-management, including features for tracking their asthma over a 12-month period rather than the 3-month period in the app, medication reminders every 24 hours at the same preselected time every day, and a calendar in the app where each day you could input…

…like what appointments you have, when, with what doctor, where, and at what time…and then have the app remind you [all within the app].

Female, 16

They also suggested a geo-mapping feature where you can plot the location at which you had an asthma attack, as well as a feature that projects the air quality for the day. Regarding the air quality checker, one adolescent stated:

If you could put some kind of air quality checker that would be really good…Just maybe put in your zip code and it could tell you like maybe just what the air quality is projected for that day, since so many people have problems with pollen and dust, and I don't know. I just thought that I would use that a lot if that was an option.

Female, 14

Many adolescents suggested adding informative asthma videos to the app. One adolescent stated:

I like the idea of having videos in the apps for people who don’t really know much about asthma and they want to learn more.

Female, 16

These informative videos would cover adolescent-recommended topics, such as peak flow, understanding asthma symptoms, and general asthma information. A video showing a medical provider was also suggested.

It has to have links to tutorials like on how to do the medication, how to work a peak flow right because that really can skew your results if you’re doing it the wrong way.

Female, 14

Like, I mean, you can look up information like reading stuff, but also like somehow put YouTube stuff, like you can like…look for videos and stuff on like how doctors and what their insight is and actually like see someone talking about it [asthma]…that’d be cool.

Female, 16

One adolescent also suggested an educational “kiddie show” for little kids with asthma, stating:

Not saying like a Sponge Bob show but…sometimes little kids don’t really pay attention to just plain old stuff, so you could put a video in there like a little kid, a kiddie show for like for asthma.

Male, 13

Furthermore, when asked, “Would you use the app to watch videos of correct inhaler technique?”, 88% (14/16) adolescents said yes. In addition, 94% (15/16) adolescents said they would take asthma quizzes if they were part of the app.

### Suggestions for Improvement and Overall Thoughts on The Apps

Adolescents also suggested other innovative features to be added to the app. These include the option to operate the app via voice command, a feature that read information in the app out to participants, a bilingual mode so individuals could engage with the app in Spanish, and an emergency button that would automatically call a contact if they were having an asthma attack. Some suggested a feature to video chat and communicate with other adolescents who have asthma as a learning opportunity to hear about how they manage their asthma, although adolescent preferences for involving friends and interacting with other adolescents who have asthma are reported elsewhere [19].

In general, adolescents responded positively to using an app for their asthma self-management needs. One adolescent stated:

I think an app can help me by like reminding me of appointments and, um, what medications to take…and organizing myself.

Female, 16

They cited that the app could help them “keep myself in check” [Male, 12], and they liked that apps allowed them to…

…really just like being able to see where you are with your asthma and you can see all of these dates at one time, and you can, you know, watch how your asthma is doing, sort of just looking at numbers and flipping through different dates. It has it all there and you can really see if it’s gone up or down or anything like that.

Female, 14

Adolescents stated that they frequently use their mobile technology for fun, and thus using apps for educational and asthma self-management purposes would easily incorporate within their daily activities.

## Discussion

### Principal Findings

Our study examined adolescent preferences and design recommendations for an asthma self-management app. Publications prior to this study have (1) elicited parent and clinician app feedback [20]; (2) documented adolescent use of apps for social support [19]; and (3) shown how existing app features positively influenced adolescent self-management via self-regulation theory constructs [21]. Adolescents additionally provided feedback on app user experience, including ease of use and new features or modifications that would encourage continued engagement. Although almost 200 asthma self-management apps exist, few comprehensive asthma self-management apps that accommodate the interests and needs of adolescents are currently available [11,22]. Our study is an important step in developing an app that optimizes the user experience by involving users in app design.

In order for a health app to be successfully adopted and regularly used, it has to be feasible or usable by an individual as part of their daily routine or in relation to a particular behavior or activity that they wish to change [23]. Adolescents in our study frequently use their mobile technology for recreational purposes and mentioned that using their devices to self-manage their asthma could be easily incorporated within their usual daily routines. Additionally, research shows that linking medication-taking with established routines may enhance health outcomes, and thus, an app that supports medication-taking and other self-management features that can be easily incorporated into their daily routine has the potential to improve health outcomes and lead to better overall asthma self-management [24].

Nearly 75% of teens play video games online or on their phone, and 47% of teens talk with others over video connections, such as Skype, Oovoo, FaceTime, and Omegle [4,10]. Therefore, our findings that adolescents are comfortable with and desire app features—including visual and auditory components—that fit within their schema, or existing frameworks of how apps should look and operate, fit well given current statistics on adolescent technology use. Our study found that although all 20 adolescents had downloaded an app, only 3 had downloaded a health-related app. Adolescents expressed that they were not accustomed to using apps like the ones in our study, and therefore, there was a slight learning curve with some adolescents expressing difficulty learning how to use the apps. To overcome this barrier, adolescents suggested video tutorials and picture tutorials that may increase their self-efficacy in using the apps.

In the context of technology adoption, research shows that self-efficacy, or the “confidence consumers have in their own abilities to understand and effectively use a new piece of technology” [25], plays a substantive role in shaping an individual’s perceived usefulness and ease of use of technology [25]. Therefore, incorporating comprehensive picture or video tutorials to show how to use the apps seems warranted to increase their perceived usefulness and ease of use of the apps, as well as subsequent asthma management. Similarly, organization and perceived visual simplicity (encompassing the use of visual aids such as pictures, graphs, and charts) were important components of the visual appeal of the apps. This is indicative of their desire for features that increase their confidence in their ability to understand and effectively use the apps, as well as engage in adequate asthma self-management. Picture and video tutorials or aids may also be beneficial for younger adolescents and those with low health literacy.

Adolescents also suggested informative asthma videos covering topics such as peak flow, understanding asthma symptoms, and general information to meet their asthma self-management needs, and 88% (14/16) adolescents said they would watch videos demonstrating correct inhaler technique. Research shows that using videos to teach inhaler technique to children with asthma can improve their inhaler technique [26,27], and thus, the incorporation of these and other informative asthma videos may increase adolescent self-efficacy in managing their asthma and lead to better health outcomes. Furthermore, adolescents in our study suggested videos showing a medical professional or age-appropriate material, as mentioned by the addition of “kiddie show” for adolescents with asthma. Research shows that adolescents and caregivers of adolescents with asthma obtain health information from health care professionals and have a high level of trust in the health care information obtained from health care professionals [28,29]. Therefore, allowing such individuals to provide clinically sound asthma information in an asthma app and directing adolescents to evidence-based asthma resources may prove beneficial. Furthermore, having peers present asthma information or appear in asthma informational shows can increase adolescent self-management efficacy through peer modeling [30,31].

Among the adolescents included in our study, 75% (15/20) said that they enjoy spending time on social networking sites, including Facebook (11/20, 55%). Some adolescents in our study suggested a feature to video chat and communicate with other adolescents who have asthma as a learning opportunity to hear about how they manage their asthma. Accordingly, 65% of the top mHealth apps connect to social media, underscoring the importance of communication features for consumer engagement [32].

In addition to including components that meet adolescent informational needs, adolescents also expressed a desire to tailor app components to meet their personal design preferences. Although perceived visual simplicity and organization were important for adolescents, mixed reviews existed on whether the adult app or child app possessed these features. Adolescents suggested modifications to the color scheme and layout (including the ability to personalize it), the inclusion of games, and other features such as the ability to incorporate Cam Newton or another person deemed inspiring by the adolescent, perhaps in an avatar format.

### Limitations

Our study has several limitations. We used a convenience sample to recruit adolescents from 2 urban pediatric clinics. Although our sample was racially diverse, we are unable to generalize our results, especially to adolescents in rural areas who may have less access to a cell phone [33]. Moreover, those who agreed to participate may have had stronger positive preferences for using technology given the iPod incentive and may have been more receptive to using an asthma app for self-management. Although 4 adolescents were lost to follow-up, they did not differ demographically from those who completed the study. Nonetheless, selection bias could have affected our results. Although participants had experience using the apps for a 1-week period, giving adolescents a longer time to explore the apps may have probably yielded additional insights.

### Conclusions

Our study found that adolescents have specific preferences for features that meet their self-management needs. App developers should consider including customizable features, such as color schemes, avatars, fonts, graphics, and sounds that fit within adolescents’ existing schema. Future research should explore whether adolescent preferences vary by gender, age, race, ethnicity, or other demographic characteristics. It is important that app developers take into consideration both adolescent feedback and evidence- and theory-based app components to ensure the development of apps that meet end-user needs and lead to improved clinical outcomes [23].

## Abbreviations

P: in-person interview
T: telephone interview
